# Arthroscopic Repair of Subscapularis Tendon Using a Percutaneous Continuous Sewing Machine–Like Suture Technique Through a Single Working Portal

**DOI:** 10.1016/j.eats.2024.103151

**Published:** 2024-08-17

**Authors:** Wenbo Yang, Yizhong Peng, Yi Li, Wei Yu, Chunqing Meng, Hong Wang, Wei Huang

**Affiliations:** Department of Orthopaedics, Union Hospital, Tongji Medical College, Huazhong University of Science and Technology, Wuhan, China

## Abstract

Tearing of the subscapularis tendon is a common shoulder injury that typically requires arthroscopic repair. The suture-passing device is a standard tool for repairing the subscapularis tendon. However, it poses the risk of device breakage and may cause additional damage to the tendon. Additionally, a traditional suture hook technique using traction sutures necessitate repeated puncturing, which increases both the complexity of the procedure and the risk of tendon damage. In this Technical Note, we describe an improvement on the previously reported continuous suturing technique by introducing a percutaneous, sewing machine–like continuous subscapularis tendon suture method through a single working portal. The key innovation in our technique is the use of a percutaneous spinal needle for suturing, along with the sewing machine–like continuous suture approach. By eliminating the traditional suture-passing devices and traction sutures, our method allows for clearer suture management; more flexible puncture site selection; and a faster, simpler suture process.

Tearing of the subscapularis tendon is a common rotator cuff injury that often requires arthroscopic treatment, with several reports of positive outcomes.^1^^,^^2^ In traditional techniques for repairing the subscapularis tendon, the use of suture passer is quite common. However, because of their large diameter and size, these devices can potentially cause additional tendon damage, especially during the process of retrograde stitching with a retrograde suture passer.^3^ Additionally, there is a risk of needle core breakage with these devices. Therefore, improvements to the traditional suture-passer technology are needed. Some clinicians have previously reported a modified technique for suturing the subscapularis tendon using a spinal needle.^3^^,^^4^ Compared with suture-passing devices, the spinal needle has a smaller diameter, minimizing the possibility of tendon damage during the suturing process. Moreover, spinal needles are easily accessible in the operating room, require no special preparation, and can achieve higher precision in suture placement during surgery. Thus, the application of the spinal needle technique in suturing the subscapularis tendon is promising. However, the suturing process of the spinal needle technique is still based on the traditional method and traction sutures are still used, thus requiring multiple punctures, suture passing, and knotting, increasing the complexity of the surgery and continuing to pose a potential risk of tissue damage. Here, based on the continuous suturing technique reported earlier,^5^ we have improved the method of suturing the subscapularis tendon with a spinal needle—namely, repair of the subscapularis tendon using a percutaneous continuous sewing machine–like suture technique through a single working portal. This technique avoids the use of traction sutures and significantly simplifies the puncture and suture-passing process. By using a sewing machine–like, continuous suturing technique, a single high-strength suture can be achieved, and multiple sutures increase the adhesion strength of the tissue, allowing more tendon tissue to be repositioned to the footprint area. This method is particularly suitable for cases with poor local tissue conditions. Additionally, unlike suture-passing devices, the spinal needle can be percutaneously punctured to reach the injury area, offering more flexibility in the choice of suturing location and clearer suture management. Theoretically, our improved subscapularis tendon suturing technique can achieve improved cost, ease, and safety.

## Surgical Technique

Our surgical protocol is shown in Video 1. The main steps are described as follows:

1. **Patient preparation and portal establishment.** After administering general anesthesia, the patient is positioned laterally with the surgical arm retracted, and a 5 kg weight is suspended from the arm. The area is then routinely disinfected and draped. The right shoulder is positioned at 50° of abduction and 15° of forward flexion (Fig 1A). First, a conventional posterior approach is created for observation, and a 30° arthroscope is used to examine the glenohumeral joint. Subsequently, the anterior operative approach is established (Fig 1A).

**Fig 1:**
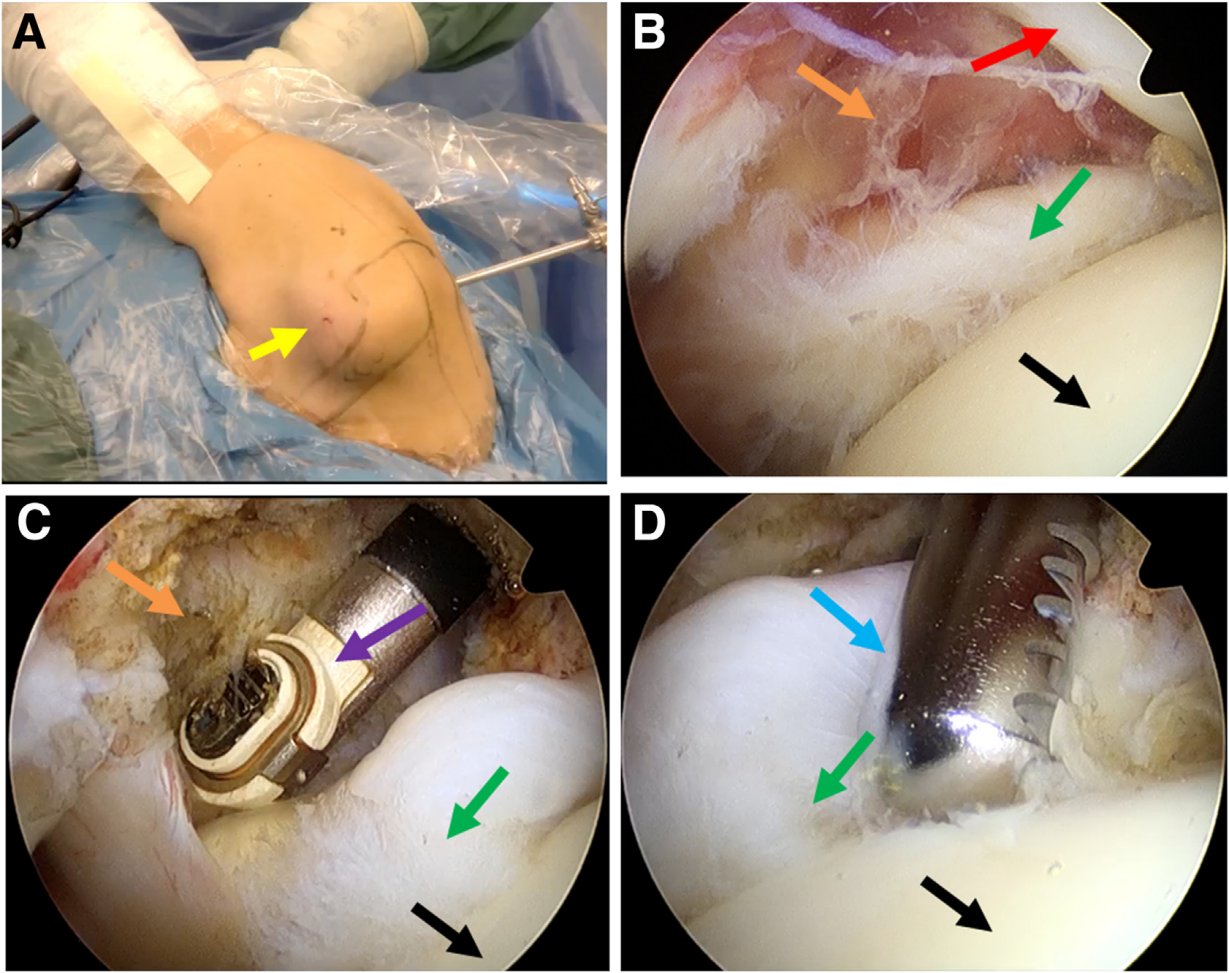
Patient preparation, portal establishment, and tear site exposure and freshening. The posterior portal is used as an observation portal. (A) The surgical position is lateral decubitus with right arm retracted. The yellow arrow indicates the anterior single working portal. (B) Tear detection. The green arrow indicates the superior third of the subscapularis. The red arrow indicates the tendon of long head of biceps brachii (LHBT). The black arrow indicates the head of humerus. The orange arrow indicates the rotator cuff interval. (C) The rotator cuff interval cleaning. The purple arrow indicates the radiofrequency. (D) Freshening of the tissue. The blue arrow indicates the shaver.
2. **Tear detection and rotator cuff interval cleaning.** The tear in the superior third of the subscapularis tendon is identified through the posterior observation portal (Fig 1B). Radiofrequency is then used to clean the rotator cuff interval (Fig 1C). After exposing the bone bed of the subscapularis footprint area, freshening is performed with a shaver (Fig 1D).
3. **The first suture: continuous sewing machine–like suture.** A 12-gauge spinal needle loaded with high-strength suture (ORTHOCORD suture; No. 2 Violet W/MO-7 1/2 CIRCLE, taper point needle, 22 mm; DePuy Mitek) is prepared. Half of the ORTHOCORD suture is left outside the needle, marked as A, and the other half is loaded inside the spinal needle, marked as B (Fig 2A). The spinal needle is inserted percutaneously below the anterior portal to penetrate the subscapularis tendon (Fig 2B); initiating the continuous sewing machine–like suture process,^5^ 3 continuous punctures are performed (Fig 3). The first puncture is made at the lowest part on the medial side of the subscapularis tear. The A end of the suture is pulled out through the anterior operation portal after this initial puncture. The A end is then pulled out again through the loop formed by the second puncture. The third puncture is made at the superior part on the medial side of the subscapularis tear, and the B end of the suture is pulled out through the anterior operation portal after this puncture.

**Fig 2:**
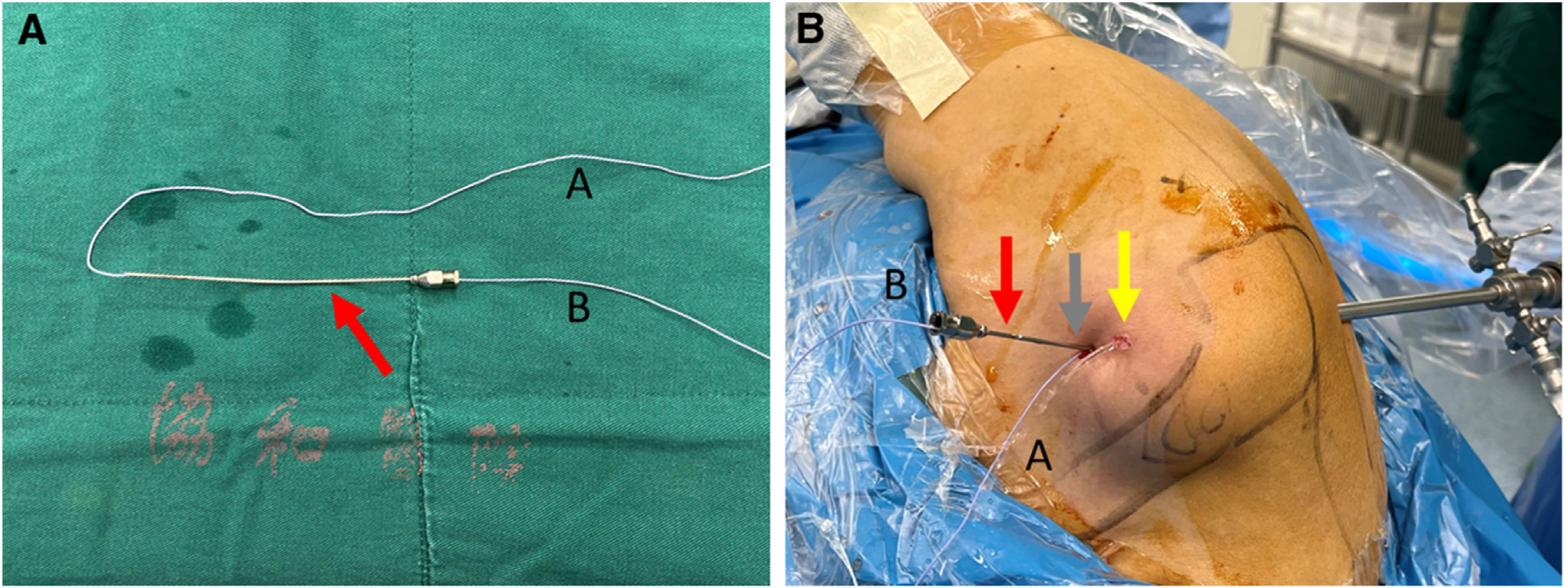
(A) The spinal needle preparation. The red arrow indicates the spinal needle. (B) The view of the spinal needle percutaneously punctured. The red arrow indicates the spinal needle, the yellow arrow indicates the anterior single working portal, and the gray arrow indicates the puncture position that below the anterior portal. In both panels, “A” indicates the part of the high-strength suture outside the spinal needle, and “B” indicates the other half inside the spinal needle.

**Fig 3:**
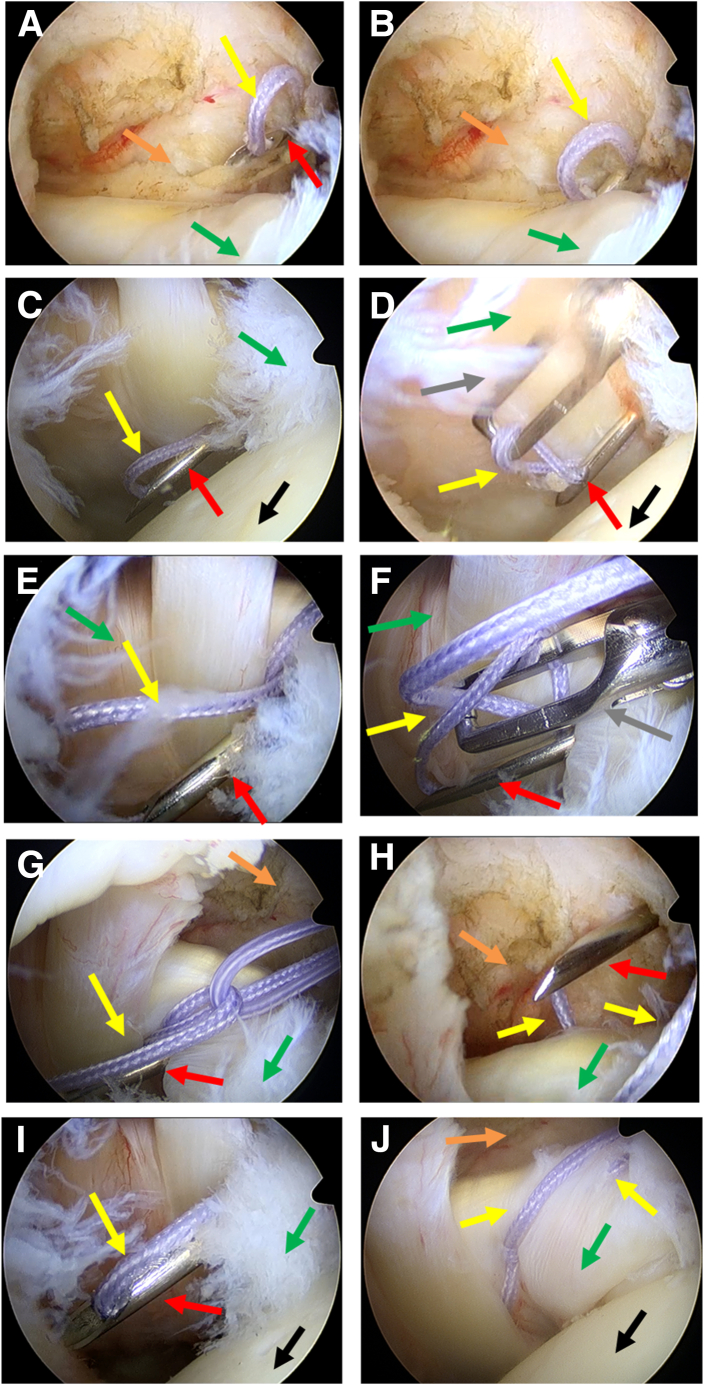
The process of the continuous sewing machine–like suture passing through the subscapularis tendon (continuous 3 punctures for the first suture). The posterior portal is used as an observation portal. (A-D) The first percutaneous puncture, which is started from the lowest part on the medial side of the subscapularis tear. Then the A end of the suture is pulled out through the anterior operation portal after the first puncture. (E–G) The second continuous puncture. The A end of the ORTHOCORD suture is pulled out again through the loop formed by the second puncture. (H–J) The third continuous puncture, which is at the superior part on the medial side of the subscapularis tear. The B end of the ORTHOCORD suture is pulled out through the anterior operation portal after the third puncture. In all panels, red arrows indicate the spinal needle, green arrows indicate the subscapularis, yellow arrows indicate the ORTHOCORD suture, orange arrows indicate the rotator cuff interval, black arrows indicate the head of humerus, and the gray arrow indicates the grasper.
4. **The second suture: one simple suture.** Another simple suture is performed with the same percutaneous suture technique. The puncture position is on the medial side of the first suture (Fig 4).

**Fig 4:**
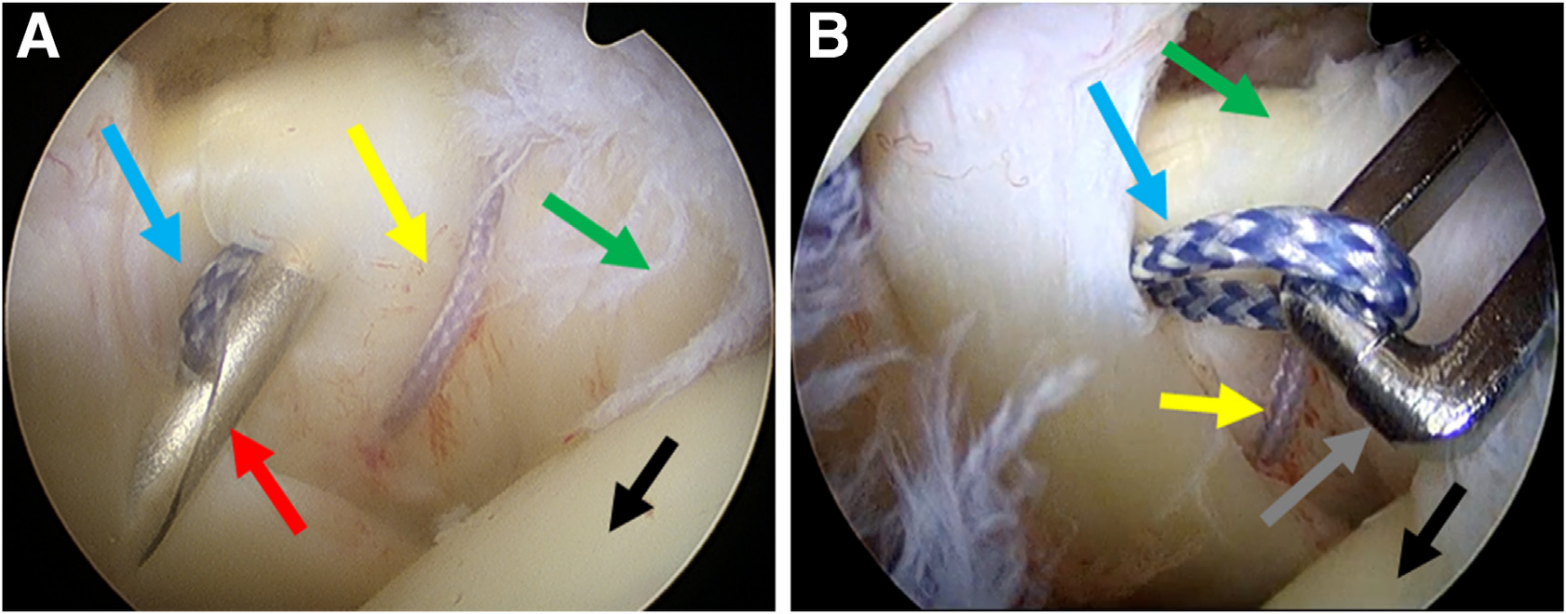
The second suture: one simple suture. The posterior portal is used as an observation portal. (A and B) The process of one simple percutaneous suture with spinal needle. Blue arrows indicate the ORTHOCORD suture used for the second suture, yellow arrows indicate the ORTHOCORD suture used for the first suture, black arrows indicate the head of the humerus, gray arrow indicates the grasper, red arrow indicates the spinal needle, and green arrows indicate the subscapularis.
5. **Single lateral row anchor implantation.** Four ends of 2 high-strength sutures are pulled out through the anterior operation portal. A single lateral row anchor (Tapscrew PK; Star Sports Medicine) loaded with all the sutures is implanted into the footprint area of the tear subscapularis (Fig 5).

**Fig 5:**
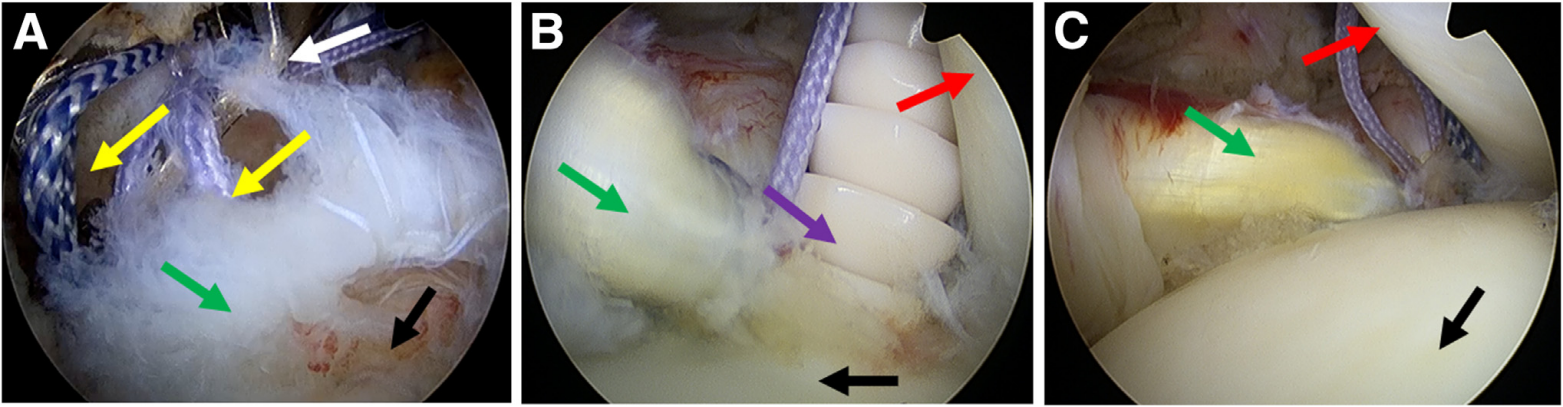
Single lateral row anchor implantation. The posterior portal is used as an observation portal (A) All suture ends are pulled out through the anterior operation portal. (B) Single lateral row anchor implantation. (C) Intra-articular observation after subscapularis repair finished. Black arrows indicate the head of the humerus, white arrow indicates the grasper, yellow arrows indicate the ORTHOCORD sutures, purple arrow indicates the lateral row anchor loaded with all the sutures, and red arrows indicate the tendon of long head of biceps brachii.

## Discussion

Arthroscopic repair of the rotator cuff is a common type of shoulder arthroscopy surgery. The tissue injury caused by repeated punctures, suture passing, and knotting during the surgery, as well as the difficulty of suture management, represents significant challenges faced by novice surgeons. Moreover, although breakage of the needle core of suture passers occurs relatively infrequently, it requires much effort to find the missing parts and may necessitate converting to open surgery, adversely affecting the patient’s prognosis. Therefore, designing simple suturing schemes that avoid the use of suture-passing devices is currently an important research direction in arthroscopic rotator cuff repair.

To our knowledge, the diameters of various suture-passing devices currently used in clinics are significantly larger than that of a spinal needle.^3^ It is possible that the application of spinal needles will reduce the risk of secondary damage to the rotator cuff. However, the previously reported rotator cuff suturing techniques using spinal needles have not solved suture management problems.^3^^,^^4^ Surgeons still need to use traction sutures skillfully and perform repeated punctures. Addressing this issue, and building on the continuous suturing technique that has been successfully applied in meniscal repair as previously reported,^5^ we now present a method for repairing the subscapularis tendon using a percutaneous, continuous sewing machine–like suture technique through a single working portal. In this technique, we use a spinal needle loaded with high-strength sutures for suturing, avoiding the use of traction sutures and making the process faster and more convenient. This method simplifies suture management, allows for more flexible selection of puncture points, and has a higher tolerance for error, making it particularly suitable for beginners.

The pearls and pitfalls of our improved technique are shown in Table 1, and the advantages and disadvantages are shown in Table 2. On the basis of case studies, our improved technique has demonstrated strong applicability and us simple, safe, and cost-effective, making it suitable for widespread adoption in arthroscopic rotator cuff repair surgeries.

**Table 1:** The Pearls and Pitfalls of Arthroscopic Repair of the Subscapularis Using a Percutaneous Sewing Machine–Like Suture Technique Through a Single Working Portal

Pearls	Pitfalls
1. The timing of consecutive punctures using the sewing machine–type continuous suture method can be determined according to the tear range of the subscapularis during the operation. Generally, 2 or 3 times are enough, but the number of times can also be increased.	1. When using the continuous sewing machine–like suture technique, the spinal needle can be withdrawn from the tendon and punctured again if the tendon puncture site is not satisfactory. However, do not withdraw the spinal needle from the skin tissue. Once the spinal needle is completely withdrawn from the skin, the continuous suturing is over.
2. The establishment of the anterior single-working approach is mainly based on the demand for implanting anchors.	2. The rotator cuff interval should be cleaned as much as possible to avoid suture management problems.
3. The percutaneous puncture position of the spinal needle is generally below the anterior approach, mainly to make it more convenient to pass through the subscapularis tendon. The spinal needle can be withdrawn and punctured again if the puncture position is not satisfactory.	
4. Cleaning the rotator cuff interval with radiofrequency before puncture is recommended. The purpose is to expose the insertion point of the spinal needle in front of the subscapularis. 5. Attention should be paid to protecting the high-strength suture to avoid cutting when the spinal needle is used to puncture. Before the grasper is used to pull out the suture, the spinal needle can be withdrawn from the tendon tissue, and then the suture can be pulled out. 6. After the suturing is complete, we recommend using a grasper to pull out the tail ends of high-strength sutures though the anterior portal to avoid interference with soft tissue and facilitate the implantation of anchor. It is also possible to use the working cannula.

**Table 2:** The Advantages and Disadvantages of Arthroscopic Repair of the Subscapularis Using a Percutaneous Sewing Machine–Like Suture Technique Through a Single Working Portal

Advantages	Disadvantages
1. In the technique, we directly use spinal needle loaded with high-strength sutures for suturing, which avoids the use of traction sutures and is faster and more convenient. 2. The single working portal is mainly for anchor implantation and thread grabbing. 3. The tendon suturing of this technique is completed through percutaneous puncture operation, which is equivalent to adding an invisible additional working portal to facilitate suture management and avoiding interference of the suturing tools and thread grabbing tools. 4. This technique uses the lateral row anchor, which can reduce the knotting process, which is faster and more convenient. 5. The continuous sewing machine–like suture technique is used. Here a single high-strength suture increases the grip of the tissue through multiple sutures and can reset more tendon tissue to the footprint area and avoid tendon tissue avulsion, which is especially suitable for tendon tissue with poor tissue conditions. 6. The working portal of this technique creates surgery positions that are more fault-tolerant and easier for beginners to master. 7. The whole process is simple, economical, and convenient, and shortens the operation time. 8. Percutaneous puncture suturing is not restricted for suturing the lower part of the subscapularis muscle. 9. The use of the lateral row anchors can avoid multiple knotting steps and achieve greater simplicity.	1. The lumbar puncture needle is directly equipped with high-strength sutures, which pose a risk of suture cutting during the suturing process and the tensioning process. 2. Transcutaneous puncture may cause damage to the traversed tissues.

## Disclosures

The authors (W.Y., Y.P., Y.L., W.Y., C.M., H.W., W.H.) declare that they have no known competing financial interests or personal relationships that could have appeared to influence the work reported in this paper.

## Supplementary Data

Video 1 Introduction of the arthroscopic repair of subscapularis using a percutaneous sewing machine–like suture technique through a single working portal.
